# A Perspective on the Use of Sexed Semen to Reduce the Number of Surplus Male Dairy Calves in Ireland: A Pilot Study

**DOI:** 10.3389/fvets.2020.623128

**Published:** 2021-02-15

**Authors:** Agnese Balzani, Cintia Aparacida Vaz do Amaral, Alison Hanlon

**Affiliations:** School of Veterinary Medicine, University College Dublin, Dublin, Ireland

**Keywords:** animal welfare, stakeholder knowledge, sexed semen, bobby calves, surplus offspring, neonatal mortality, veal calves, bull calves

## Abstract

The production of surplus male offspring illustrates a socioethical concern in the dairy industry. In this article, we highlight the animal health and welfare implications of production outputs for surplus dairy calves, namely veal production, dairy calf to beef production, and euthanasia. Moreover, we present a pilot study focus on exploring the perception of key industry actors within the dairy industry in Ireland regarding the use of sexed semen as a mitigation strategy to reduce the production of surplus male dairy calves. A pilot survey was completed by farmers (*n* = 6), veterinarians (*n* = 17), and dairy farm advisors (*n* = 11). All the veterinarians, 80% of the farmers, and 62% of the advisors believed that the use of sexed semen had a positive influence on herd welfare. All participants identified the same barriers to the implementation of sexed semen: lower conception rate, lower availability, and higher cost. The reviewed literature highlights the importance of tailored communication to support knowledge exchange between stakeholders and key industry actors such as dairy farmers, their veterinarians, and advisors. Research to understand stakeholders' perception is pivotal to address socioethical concerns such as the surplus male dairy calves.

## Introduction

The increased demand for animal products led to the industrialization of the agriculture system in the northern hemisphere in the mid-nineteenth century, in particular milk production has increased exponentially per farm, per cow, and per input of feed and labor (1). Retailers' control of milk supply chains has enabled supermarkets to sell milk below production cost to attract customers (1). Inevitably, the low retail pricing has put dairy farmers under financial pressure, which has been a driver to herd expansion for farmers to remain competitive (2). Following the abolition of milk quota in the European Union, dairy herd expansion was encouraged by the Irish government and supporting research bodies. To maintain economic sustainability, dairy farmers in Ireland followed this guidance to expand their herd (3).

Between 2015 and 2016, milk production in Ireland increased by 18.5%, supported by an increase in the number of dairy cows by 23% from 2013 to 2017 (4). Irish dairy farming is seasonally grassland-based, providing a lower cost production system by maximizing pasture utilization. Compact calving in the spring is a key strategy to support production efficiency and is characterized by a 6-week calving period. Compact calving has increased from 61 to 72%, and the mean calving date has advanced from the 11th to the 3rd of March between 2008 and 2017; by 2018, 84% of calvings occurred between January and April (compared with 74% in 2008) in Irish dairy herds (5). Such technical successes have been achieved in spite of ongoing labor challenges (5).

At the same time, there is growing political emphasis on environmental and social sustainability of agriculture, and farmers are seen as the actors best positioned to improve their sustainability credentials and safeguard animal health and welfare, which may in the short-term impact economic returns (6). Animal welfare (AW) is an ethical concept and is subject to societal input (7). The management of surplus male dairy calves is an emblematic example of the ethical context of AW (7). The low economic value of male dairy calves, of Jersey and Jersey-cross sires influences the production outcomes. In Ireland, there are three main outcomes for surplus male dairy calves: export to continental Europe for veal production, dairy to beef production, and euthanasia. Societal concerns have been expressed, in the national and international media, regarding live exports and euthanasia of young calves. Alternatives to the production of low-value surplus male dairy calves are required to mitigate against reputational risks to the industry and the corresponding social license to operate. Such alternatives may include novel breeding or selection methods, the use of less-specialized breeds which may have additional advantages, such as improved resilience, or rearing animals for special markets (8). For example, embryo sex predetermination has been a goal of the beef and dairy production system since its industrialization (9). Despite being available since 1990s, this technique is not widely used, particularly in Ireland where sexed semen needs to be imported (8). Most of the research on sexed semen deals with technical aspects of sex determination and breeding of dual-purpose breeds, and there is little on the knowledge and preferences of actors working in the dairy sector regarding this alternative solution. To date, only a limited number of stakeholders' views on surplus male dairy calves have been identified (10), and currently, there is no evidence on industry actors' views on strategies for avoiding surplus male dairy calf production in Ireland. This indicates a need to understand the various perceptions of sexed semen that exist among industry actors namely, farmers, veterinarians, and advisors.

To move away from the production of surplus offspring, the Federation of Veterinarians of Europe highlighted the need to promote key stakeholders' collaboration to address and design new solutions (11). In this regard, understanding stakeholders' views and values of AW concepts can lead to more effective development of collaborative knowledge exchange, policies, and management of initiatives directed at improving AW, socially accepted, and thus sustainable livestock farming practices (12, 13). Our thesis is that industry actors play an important role for the implementation of an alternative solution to surplus male dairy calves. We use a pilot study to illustrate the AW implications of surplus male dairy calves, factors that influence the use of sexed semen and show how the perception of actors in the dairy sector in Ireland influences the decision-making process.

## Animal Welfare Implications for Surplus Male Dairy Calves

**Figure 1:**
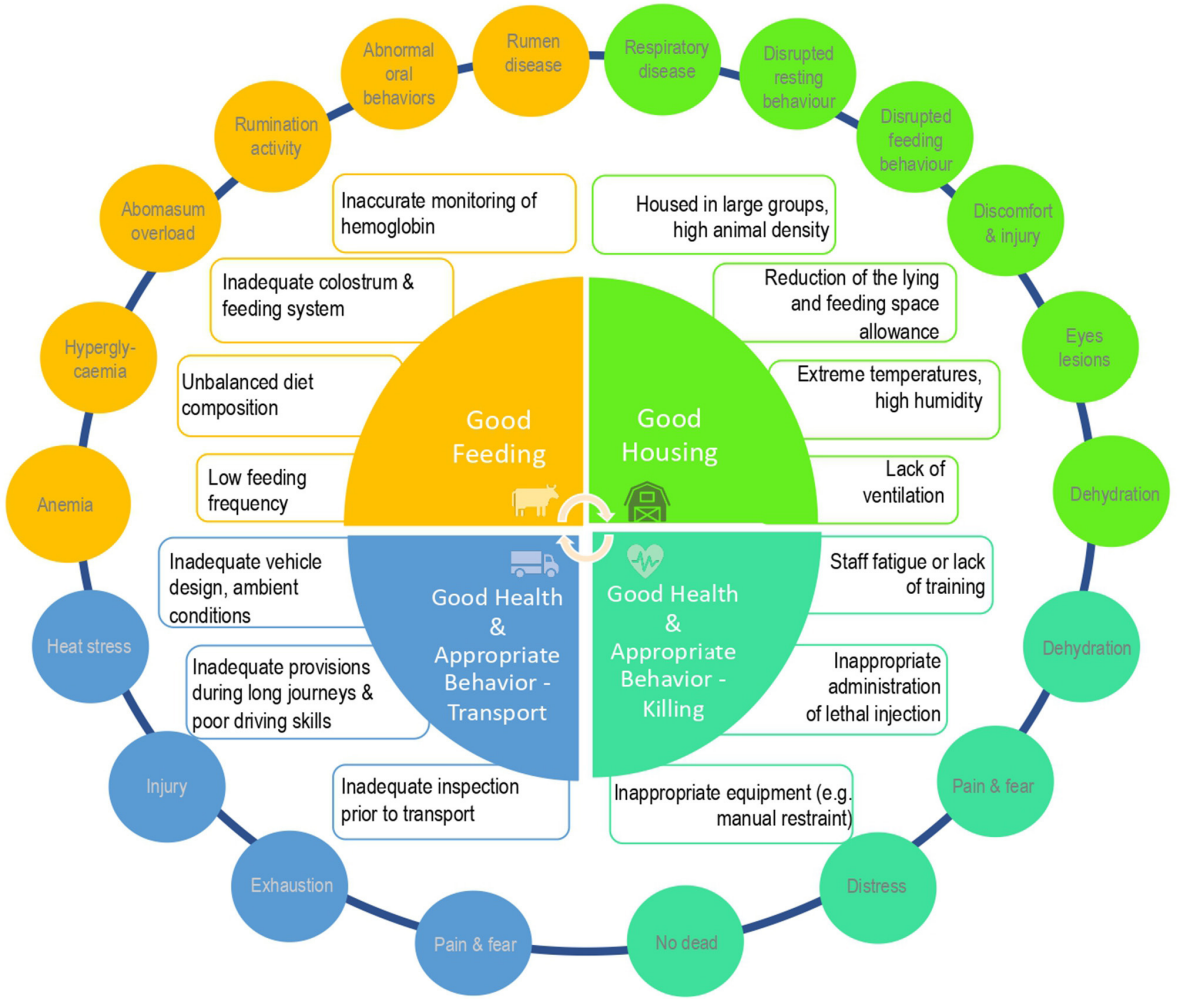
Summary assessment of the animal welfare (AW) outcomes of young male dairy calves during transport (blue frame) and killing (light green frame), modified from Welfare Quality's® four principles: Good Feeding (yellow frame), Good Housing (green frame), Good Health, and Appropriate Behavior (light green and blue frame). List of main hazards (in the internal rectangular boxes) and welfare outcomes (in the external circles) in young male dairy calves.

Unlike female dairy calves, which can be reared as replacement heifers, male dairy calves, especially of Jersey and Jersey crossbreed, are surplus to requirements and depending on the breed may be unsuitable for beef production. Dairy farmers in Ireland are currently faced with three main options: live export for veal production (production of food not for human consumption, such as pet food), calf to beef production (excluding Jersey and Jersey crossbreeds), or euthanasia (14). Figure 1 shows a summary assessment of the main AW outcomes for surplus male dairy calves by modifying the Welfare Quality® Four Principles of Good Feeding, Good Housing, Good Health, and Appropriate Behavior during transport and at killing (15).

### Veal Production or Bobby Calves

There is no viable veal production industry in Ireland. Male dairy calves destined for veal production in continental Europe are exposed to stressors during marketing and transport within 2 weeks of birth (8). When animals are sold through markets, travel time and fasting may be prolonged, and grouping with unfamiliar animals is increased presenting significant hazards to calf health and welfare (16). In New Zealand, calf mortality was reported at 0.12% in 2016 (17). Long distance transport of young male “bobby” calves in Australia to slaughter plants was associated with approximately 0.6% mortality during transport due to adverse environmental stressors (18). In Ireland, calves are exported by road, sea, and air to continental Europe for veal production (19). Irish animals travel to the Continent on roll-on, roll-off ferries direct from Ireland to France (a sea journey of around 18.5 h) (19).

### Dairy Calf to Beef Production

Dairy to beef production, which utilizes male dairy calves, has been reported to be a more efficient way of producing beef (20). Approximately 50% of beef in the UK originates from the dairy herd (21), 18% of dairy calves are raised as young bulls in France, and 20% are crossbred between a dairy dam and a beef sire (22). While research from Denmark on dairy calf to beef production has encouraged farmers to increase the use of beef breed sires by approximately 20% (23), currently, it accounts for 12% and 8% in Sweden (24).

### Euthanasia

The killing of surplus offspring might not be an AW issue *per se*, provided that the killing is performed humanely following best practice (25). There are several AW risks with on-farm euthanasia, for instance the application of inadequate killing methods or the treatment of the animals before killing, particularly since they lack economic value (26). In addition to AW considerations, the killing of surplus male livestock raises moral concerns as the animal is seen as an surplus “by-product” or waste, which disrespects its intrinsic value (26).

## Sexed Semen as an Alternative Solution

Flowcytometric separation of X- and Y-bearing semen (sexed semen) was first adopted for cattle breeding in 1989 (27). The main purpose of this technology is for production of higher genetic merit heifers. Despite the technology advancements including 90% female determination (8, 28), genetic gain (29), and reduced incidence of dystocia (8), the implementation of sexed semen on-farm is influenced by the reduced conception rate (CR) and the additional cost (€38 vs. €18 sexed vs. conventional semen in Ireland) compared with conventional artificial insemination (8).

### Industry Actors' Knowledge on Sexed Semen Use—Pilot Study

A pilot survey was completed online by farmers (*n* = 6), veterinarians (*n* = 17), and advisors (*n* = 11) involved in dairy production in Ireland. Data were collected *via* an anonymized online web-based survey distributed by representative organizations involved in the dairy sector, using snowball techniques. A draft of the survey was peer reviewed by two national experts in genetics and breeding prior to finalization. Three surveys were developed, designed for each industry actor's category to explore the perception of the use of sexed semen and its potential to support dairy cow and calf welfare. The surveys were structured in four parts: (i) general information about farming experience, herd size, and frequency of sexed semen use, (ii) the advantages of using sexed semen, for example, for AW, biosecurity, productivity, to reduce male calves, and to increase heifer calves born; (iii) the disadvantages of sexed semen, for example, commercial and breed availability, price, and low conception rates (CR); and (iv) the perceptions of the use of sexed semen including knowledge (cost, CR, use) and social influencers (media, advisors, veterinarians, producers).

## Results

Experience working in the dairy sector varied with industry actor's category. Veterinarian respondents had on average 8 years in practice (range 3 to 23 years), advisors 15 years (range 1 to 32 years), and farmers had 32 years (range 12 to 43 years) of experience. Herd size of veterinarians' clients ranged from a mean small herd size of 27 animals (range 12 to 50) to a mean large herd size of 689 animals (range 250 to 2,000). The advisors' clients ranged from a mean small herd size of 37 animals (range 20 to 70) to mean large herd size of 524 animals (range 250 to 1,000). Farmers had a mean herd size of 135 animals (range 70 to 222). All the farmers questioned had increased their herd size since the end of milk quota by an average of 45 animals (range 15 to 92). Table 1 summarizes the survey's responses.

**Table 1:** Survey responses (expressed in percentage and euro) of farmers (*n* = 6), veterinarians (*n* = 17), and advisors (*n* = 11) involved in dairy production in Ireland, to explore the knowledge and perception on using sexed semen to reduce surplus male offspring.

**Perceptions**^**a**^	**Factors**	**Farmers** ** (%)**	**Advisors** ** (%)**	**Veterinarians** ** (%)**
Advantages	Increases heifer calves born	67	100	87
	Reduces male calves born	100	88	73
	Increases productivity	33	25	53
	Improves biosecurity	0	12	27
	Improves cost benefit	20	38	87
	Improves herd welfare	80	67	100
Disadvantages	Conception rates	100	100	86
	High costs	83	50	73
	Breeds availability	33	75	75
	Commercial availability	17	13	40
Knowledge	Costs	46 euro	38 euro	35 euro
	Heifer use	100	38	20
	Conception rate	61	60	64
Social influencers	Producers	50	67	38
	Veterinarians	0	27	15
	Advisors	50	67	75
	Media	0	33	13

a *Questions asked about sexed semen technology were as follows: Advantages: it increases heifer calves born; it reduces male calves born; it increases productivity; it improves biosecurity; it improves cost-benefit; it improves herd welfare. Disadvantages: it reduces conception rates; it has high costs; not many breeds available; it is not commercially available. Knowledge: how much sexed semen cost; what is the desired use of sexed semen; what is the conception rate using sexed semen. Social influencers: producers influence the use of sexed semen; veterinarians influence the use of sexed semen; advisors influence the use of sexed semen; media influence the use of sexed semen*.

All the veterinarians (17/17), 80% of the farmers (5/6), and 62% of the advisors (7/11) believed that the use of sexed semen had a positive influence on herd welfare. Veterinarians researched the use of sexed semen more than advisors or farmers and commented on the benefit to AW of using sexed semen to improve calving, for example, “*heifers have easier parturition and smoother transition to lactation*,” more time for calves, for example, “*A farmer will always (sometimes subconsciously) look after heifer calves better*,” and farmer labor “*as it improves farm efficiency then it decreases farm workload etc. which would have a positive knock-on effect on welfare*.” Advisors who commented on the positive impact of sexed semen often used an economic frame of reference, for example, “*it may have some effect if the calves for sale are higher in value then they may be looked after better vs. a low value calf destined for sale*,” “*in theory more heifers and less bulls but conception rates have been low*,” and “*there is enough easy calving sires available through conventional semen*.”

When asked general questions about use, cost, and conception rate, farmers estimated that average sexed semen conception rate was 61% (range 50 to 76%), advisors estimated 60% (range 50 to 85%), and veterinarians 64% (range 50 to 80%). Performance was a determinant of whether to use sexed semen, for example, “*I don't agree with using it on heifers because you could be breeding the next generation from unproven animals*” (veterinarian), “*conception rates are low normal around 40%. Top bulls not available*” (advisor).

The average cost of a straw of semen was estimated at €38 (range 25 to 48 euro) by advisors, €35 (range 14 to 60 euro) by veterinarians, and €46 (range 38 to 60 euro) by farmers. A smaller proportion of advisors (4/11) and farmers (1/6) believed that sexed semen justified the investment, whereas 87% of the veterinarians (15/17) agreed that the benefits of sexed semen justified the investment. A key benefit referred to by industry actors was genetic gain; for example, “*Once the conception rates mirror those of normal (non-sexed) semen then it is a no-brainer for dairy farmers as a tool to improve farm productivity*” (advisor), “*more renewal of herd with less losses in male dairy calves and quicker genomic selection*” (advisor), “*increase productivity of dairy farms*” (veterinarian). In contrast financial issues were a disincentive, for example, “*due to expansion of herd size and associated costs many farmers cannot allocate the time and resources*” (advisor), “*if conception rates are low due to sexed semen, then it can create knock on problem for the milking herd, for example, loss of production with lower number of cows calving down in the first 3 weeks*” (advisor).

When asked about knowledge on the use of sexed semen, advisors (7/11) and veterinarians (14/17) indicated a wider use whereas farmers (6/6) reported that they would only use sexed semen as a replacement strategy for heifers. Farmers (4/6) used sexed semen but mainly in heifers, often for performance reasons, for example, “*used in strong heifers, very poor results in cows*” (advisor), “*only heifers perceived as too expensive with low conception rates in*” (veterinarian).

When asked who or what was the main influencer for farmers deciding to use sexed semen advisors believed that they were the main social influencer (75%; 7/11) followed by salespersons (50%; 5/11, i.e., AI tech companies). The majority of the veterinarians believed that both peers (67%; 10/17) and advisors (67%; 10/17) were the main influencers for farmers. Few veterinarians believed that they influenced the farmer's decision (20%; 4/17) and indicated that salespersons had a greater influence (33%; 5/17). Farmers recognized their peers (50%; 3/6) and advisors (50%; 3/6) as having the main influence on their decision to use sexed semen. Farmers also identified their own research activities as the major influence on their decision (80%; 4/6).

## Discussion

This pilot study presents a perspective on the knowledge and understanding of key actors in the Irish dairy sector on the application of sexed semen as a potential mitigation strategy to reduce the production of surplus male dairy calves. Production outputs for male dairy calves, depending on the breed, include dairy calf to beef, veal production and, where there are no cost-efficient alternatives, euthanasia. In Ireland, as there is no viable veal industry, calves are exported live to continental Europe, used in dairy calf to beef, and or euthanized. Whatever the production output, it is essential that calf welfare standards are maintained.

The pilot survey identified that sexed semen was used mainly to improve genetic gain, it showed concerns over reduced pregnancy rates in cows and preferential use in heifers, findings which are in agreement with Holden and Butler (8). The key determinants for the survey participants were the lower conception rates achieved with sexed semen compared with conventional semen and lack of availability of sexed semen from high genetic merit sires in Ireland. The barriers to using sexed semen identified in this pilot study were similar to Johnson (9) even if recent evidence asserted that sexed semen can be successfully used in both heifers and multiparous cows, high-value bull semen is widely available in most of the developed countries, and methods of extended semen storage, particularly for liquid semen are in place (28, 29).

Moreover, the pilot survey's respondents perceived the cost of sexed semen as a barrier. Interestingly, veterinary practitioners and farm advisors estimated the cost to be higher than farmers. In-depth interviews with Swedish farmers revealed a number of concerns regarding the use of sexed semen for production of higher genetic merit heifers, namely lower conception rates, more difficult calving and increased stillbirths, an increased risk of disease transmission, and that sexed semen was considered were more time consuming and could lead to poorer animal welfare, such as calving difficulties and diseases (30). Some of the perceptions by Swedish farmers are not based on current evidence and demonstrate suboptimal dissemination of research findings and knowledge exchange. Other studies have reported asymmetrical perception (31) and poor communication (32) between farmers and their veterinarians.

Knowledge and tailored communication for effective understanding between the farmer and veterinarian were the most important factors influencing uptake of AW innovations (33). Undoubtedly, knowledge empowers people in decision-making and behavioral change. Since the late 1980s, Hemsworth (34) showed a positive correlation between knowledge and willingness to improve AW.

Lack of knowledge has been identified as the cause of failure to recognize problems, also referred to as farm blindness “a misperception by farmers that what they see every day on their own farm is normal” (35). In this regard, farm blindness is less likely to happen when farmers engage in knowledge dissemination, for example, farming discussion groups (36).

While the majority of each industry actor cohort agreed that sexed semen could positively impact AW, only veterinarians believed that the benefits of sexed semen justified the investment to improve AW. The financial costs of implementing improved AW practices were identified as a key external factor influencing farmers' perspectives of AW by Balzani and Hanlon (12). However, the issue of management of surplus male dairy calves goes beyond external costs, because of the implications for reputation risk to the industry. Accounting for reputational risk into a cost-benefit analysis may provide an important decision-making tool to support the implementation of sexed semen to reduce the number of surplus offspring.

The pilot survey explored industry actors' perceptions of key influencers. There is increasing evidence about models of communication between scientists, veterinarians, advisors, and farmers. Indeed, Vigors (37) reported that “the words used to communicate AW to non-specialists may be more important than knowledge of welfare itself.” Outcomes of 110-veterinarians and 116 farmers interviews about dehorning cattle showed that veterinarians had a poor understanding of farmers' priorities, that affected their guidance on methods to improve calf welfare (38). In this context understanding farmers' goals was a key skill for veterinary practitioners to encourage improvement of AW (39). Furthermore, a review on farmers' and veterinarians' attitudes toward cattle welfare showed that the farmer–veterinarian cooperation reduced barriers to improvements in dairy cattle welfare, achieved by identifying shared concerns about AW, reframing their unique perspectives as complementary roles, and promoting communication about priorities and goals (40).

Several authors concluded that beliefs and acceptance among farmers may be influenced through a communication strategy (33, 35, 41). Purwins and Schulze-Ehlers (42) suggested that there may be a value in facilitating positive word-of-mouth. The same view has been proposed by Horseman (43) of farmers sharing their positive experiences on handling facilities, and by Hennessy and Heanue (36) testing farmer action and discussion groups for effective peer-to-peer learning. Duval (44) used a participatory approach to enhance knowledge exchange between farmer and scientist.

### Study Limitations

This pilot study served to explore perception and knowledge among dairy farmers, farm advisors, and veterinarians in Ireland regarding sexed semen as a mitigation strategy to address surplus male dairy calves. The authors acknowledge that the pilot study contained a small and imbalanced sample size. However, the insight provided by the pilot survey serves as a guide for further investigation. Follow-on studies would benefit from engaging with more stakeholders including the processors and consumers.

## Conclusion

The use of sexed semen will not entirely eliminate the problem of surplus male dairy calves, as other strategies are needed to reduce the numbers of surplus female dairy calves. The use of beef sires in dairy herds would produce both male and female calves that have a higher value for the beef and veal markets (11). According to Seidel (20), beef production is on the cusp of change, and by 2050, he envisaged that beef sucklers will be replaced by the use of sexed semen, and the establishment of dairy calf to beef systems. This shift in the beef industry would reduce the GHG emissions (45), partially addressing societal concerns regarding surplus dairy calf production and climate change (8).

While sexed semen is a promising mitigation strategy, its implementation may elicit societal ethical concerns that are underexplored (46). However, the alternatives to sexed semen such as live export of young calves for dairy-beef or veal production and euthanasia, may also conflict with societal views (47). Ethical analysis of livestock production science is increasing, providing an important contribution to the development of policy and practice (48). Farmers have been left in a difficult position, and societal debate and greater stakeholder engagement is required to support major changes in policy such as dairy herd expansion and the *socioethical concern* of surplus male dairy calves (49).

Whether mitigation strategies are adopted will depend on resources, the interest, and knowledge of industry actors and other stakeholders. Therefore, stakeholders' perception is pivotal to understand barriers to behavioral change and supporting AW innovations. Further research is required to engage the full spectrum of stakeholders to identify the most effective means of strategic support for dairy farmers to reduce the production of surplus calves.

## Data Availability Statement

The raw data supporting the conclusions of this article will be made available by the authors, without undue reservation.

## Ethics Statement

The study complied with UCD Research Ethics Guidelines and qualified for exemption from full ethical approval by the University College Dublin Human Research Ethics Committee (UCD HREC) (LS-E-18-151-Hanlon). The participants provided their informed consent to participate in this study.

## Author Contributions

The survey was designed by AH and CA. CA performed the data collection. AB performed the data analysis for the manuscript and developed the first draft of the manuscript. AH reviewed and amended the manuscript during its development. All authors contributed to the article and approved the submitted version.

## Conflict of Interest

The authors declare that the research was conducted in the absence of any commercial or financial relationships that could be construed as a potential conflict of interest.
